# National ambulatory care non-insulin antidiabetic medication prescribing trends in the United States from 2009 to 2015

**DOI:** 10.1371/journal.pone.0221174

**Published:** 2019-08-14

**Authors:** Amanda K. Kitten, Meghan Kamath, Laurajo Ryan, Kelly R. Reveles

**Affiliations:** 1 College of Pharmacy, The University of Texas at Austin, Austin, Texas, United States of America; 2 Pharmacotherapy Education and Research Center, The University of Texas Health San Antonio, San Antonio, Texas, United States of America; Mahidol University, THAILAND

## Abstract

**Objective:**

Despite their efficacy in lowering hemoglobin A1c, recent data suggest that sulfonylureas are associated with cardiovascular risk and hypoglycemia. The objective of this study was to determine whether prescribers decreased sulfonylurea use in favor of newer medications in the United States over seven years.

**Research design and methods:**

This cross-sectional study utilized data from the Centers for Disease Control and Prevention’s National Ambulatory Medical Care Survey. Patient visits between 2009 and 2015 were included for patients who were at least 18 years old, had a documented prescription for a non-insulin antidiabetic medication, and a diagnosis of type 2 diabetes. Sample survey data were extrapolated to national estimates using data weights. Prescribing rates were calculated as the number of visits with a documented medication class divided by the total number of visits with a prescription for any diabetes medication class, times 100%.

**Results:**

A total of 303 million patient visits were included in this study. The median (IQR) patient age was 64 (55–73) years old and 49.8% were male. Sulfonylurea prescribing rates decreased from 43% in 2009 to 36.5% in 2015. Prescribing of GLP-1 receptor agonists increased from 2009 to 2014 (3.95% to 5.30%), but then decreased to 4.19% in 2015. SGLT-2 inhibitor prescribing began in 2013 and increased to 7.3% by 2015. Metformin prescribing remained relatively stable over the study period (range 70% to 72%).

**Conclusions:**

National ambulatory sulfonylurea prescribing decreased from 2009 to 2015 with a corresponding increase in newer non-insulin antidiabetic agent prescribing.

## Introduction

Diabetes is a growing public health concern in the United States (U.S.). Approximately 30.3 million people (9.4% of the U.S. population) had diabetes in 2017 [1]. Of these diagnoses, 90–95% were for type 2 diabetes. Diabetes complications, including cardiovascular disease, lower limb amputations, and diabetic ketoacidosis, result in significant costs to the healthcare system—approximately $407 billion in 2015 [2].

Type 2 diabetes treatment options have expanded markedly over the last 20 years [3]. Of these new agents, the glucagon-like peptide-1 receptor agonists (GLP-1 RAs), sodium-glucose co-trasport-2 (SGLT-2) inhibitors, and dipeptidyl-peptidase-IV (DPP-IV) inhibitors are recommended by the American Diabetes Association (ADA) alongside thiazolidinediones (TZDs) and sulfonylureas as options for dual therapy with metformin, or first line therapy when metformin is contraindicated [4].

Of the non-insulin antidiabetic agents recommended by the ADA, sulfonylureas have been in use for the longest [3]. Despite their ability to significantly lower hemoglobin A1c (HbA1c) levels, a growing body of evidence over the last decade suggests that sulfonylurea use in the treatment of type 2 diabetes can be harmful to patients for several reasons [3]. Their use has been associated with cardiovascular mortality [5], and they are likely to cause hypoglycemia which can result in falls and fractures [6, 7]. Conversely, the novel antidiabetic agents provide substantial advantages; for example, some SGLT-2 inhibitors and GLP-1 RAs may be of particular benefit to patients with type 2 diabetes due to their propensity to cause weight loss and provide cardiovascular benefits [8–11]. They also help repair the dysregulated pathways that contribute to the pathophysiology of type 2 diabetes [7].

With new agents and their potentially advantageous side effect profiles, the heath care community can expect movement away from sulfonylurea prescribing in favor of these newer agents. The objective of this study was to examine national sulfonylurea prescribing trends in the U.S. in relation to other non-insulin antidiabetic agents over a seven year period.

## Research design and methods

### Study design and data source

This was a cross-sectional study utilizing the Centers for Disease Control and Prevention’s (CDC) National Ambulatory Medical Care Survey (NAMCS) from 2009 to 2015. NAMCS is a national, annual survey of a sample of visits to non-federally employed, office-based physicians engaged in direct patient care [12]. NAMCS was designed to provide objective, reliable information regarding the provision of ambulatory medical care services in the Unites States. The survey collects major reason for visit, chronic problems, injuries, new problems, preventive care, and medication information. At least 3 diagnoses and at least 8 medications are collected per patient visit. Beginning in 2014, physicians were able to include up to 30 medications per visit; however, we utilized the first 8 listed medications from each year to ensure consistent risk for misclassification of data across all years. Previous publications have discussed in detail the data collection methods and definitions used in the NAMCS [13, 14].

### Study population

We included outpatient visits between 2009 and 2015 for patients at least 18 years of age with a documented prescription for at least one non-insulin antidiabetic medication class, including: biguanides (e.g. metformin), sulfonylureas, TZDs, GLP-1 RAs, SGLT-2 inhibitors and DPP-4 inhibitors. Patient visits that did not document diabetes as a current disease state or include an ICD-9-CM code consistent with type 2 diabetes (250.XX) were excluded. The antidiabetic drug classes were chosen based on the ADA Standards of Care 2018 treatment algorithm. Medications were identified using Multum codes. Combination medications were included in both medication classes for analysis.

### Statistical analysis

The survey data were analyzed using assigned sampled visit weights adjusted by the National Center for Health Statistics to extrapolate to national estimates. Sulfonylurea and other antidiabetic agent prescribing rates were determined by dividing the number of visits with a documented prescription for each designated medication class by the total number of visits with a documented prescription for any of the above listed antidiabetic classes times 100%. Age, payer, sex, ethnicity, race, heart failure status, coronary artery disease, obesity, and region were compared between patient visits with a documented sulfonylurea prescription and those without using the Wilcoxon rank-sum test for continuous data and the chi-square test for nominal data. Overall sulfonylurea prescribing rates were analyzed by year for the overall population and in the elderly (older than 65 years), Hispanic and Latino, African American, and private insurance populations. Rates of prescribing of the novel non-insulin antidiabetic agents were also analyzed by payer. A logistic regression model was performed to identify patient characteristics predictive of sulfonylurea use. Model covariates included year, region, age, sex, ethnicity, race, payer, heart failure, coronary artery disease, and obesity.

## Results

A total of 303 million patient visits were included in this study. Overall, the median (IQR) age of patients included was 64 (55 to 73) years, and the number of males and females was approximately equal at 151.1 million and 152.2 million, respectively (Table 1). Overall, the total number of prescriptions from 2009 to 2015 was highest for metformin (217 million, 72%) followed by sulfonylureas (123.5 million, 41%). Prescriptions for all other non-insulin agents combined comprised less than 50 million per year. With respect to payer, private insurance and Medicare paid for the majority of all non-insulin antidiabetic agent prescriptions at 39% and 45.3%, respectively. Subjects prescribed a sulfonylurea were older than those without sulfonylurea prescriptions (67 versus 62 years). In a multivariable mode, the strongest predictors of sulfonylurea prescription were heart failure (OR 1.57; 95% CI 1.56–1.57; p<0.0001), age greater than or equal to 65 years (OR 1.53; 95% CI 1.53–1.54; p<0.0001), male sex (1.24; 95% CI 1.24–1.24; p<0.0001), and African American race (OR 1.20; 95% CI 1.19–1.20; p<0.0001).

**Table 1:** Population characteristics.

Characteristic	Overall prescriptions (millions)	Sulfonylurea prescription (millions)	No sulfonylurea prescription (millions)	P-value
Age (years), median (IQR)	64 (55–73)	67 (58–76)	62 (54–71)	<0.0001
Private insurance (%)	119 (39%)	41.5 (33.6%)	77.9 (43.3%)	<0.0001
Medicare (%)	137.3 (45.3%)	63.2 (51.2%)	74.1 (41.2%)	<0.0001
Female sex (%)	152.2 (50%)	58.4 (47.3%)	93.8 (52.2%)	<0.0001
Hispanic or Latino	37.1 (12.2%)	14.2 (11.5%)	22.9 (12.7%)	<0.0001
Race				<0.0001
American Indian	1.3 (0.6%)	0.46 (0.2%)	0.84 (0.37%)	
Asian	12.1 (5.3%)	5.4 (5.7%)	6.7 (5%)	
African American	37.5 (16.4%)	15.6 (16.5%)	21.9 (16.3%)	
Pacific Islander	1.0 (0.45%)	0.5 (0.53%)	0.5 (0.39%)	
White	177 (77%)	72.5 (77%)	104 (78%)	
HF	12 (4.0%)	6.4 (5.19%)	5.6 (3.1%)	<0.0001
CAD	36.1 (11.9%)	16.8 (13.6%)	19.3 (10.8%)	<0.0001
Obesity	65 (21.5%)	26 (21.1%)	39 (21.8%)	<0.0001
Region				<0.0001
Midwest	64 (21.1%)	27.2 (22.1%)	36.7 (20.5%)	
Northeast	53.7 (17.7%)	21.3 (17.2%)	32.4 (18%)	
South	114 (37.7%)	46 (37.2%)	68.1 (38%)	
West	71.2 (23.5%)	29 (23.5%)	42.2 (23.5%)	

**Fig 1:**
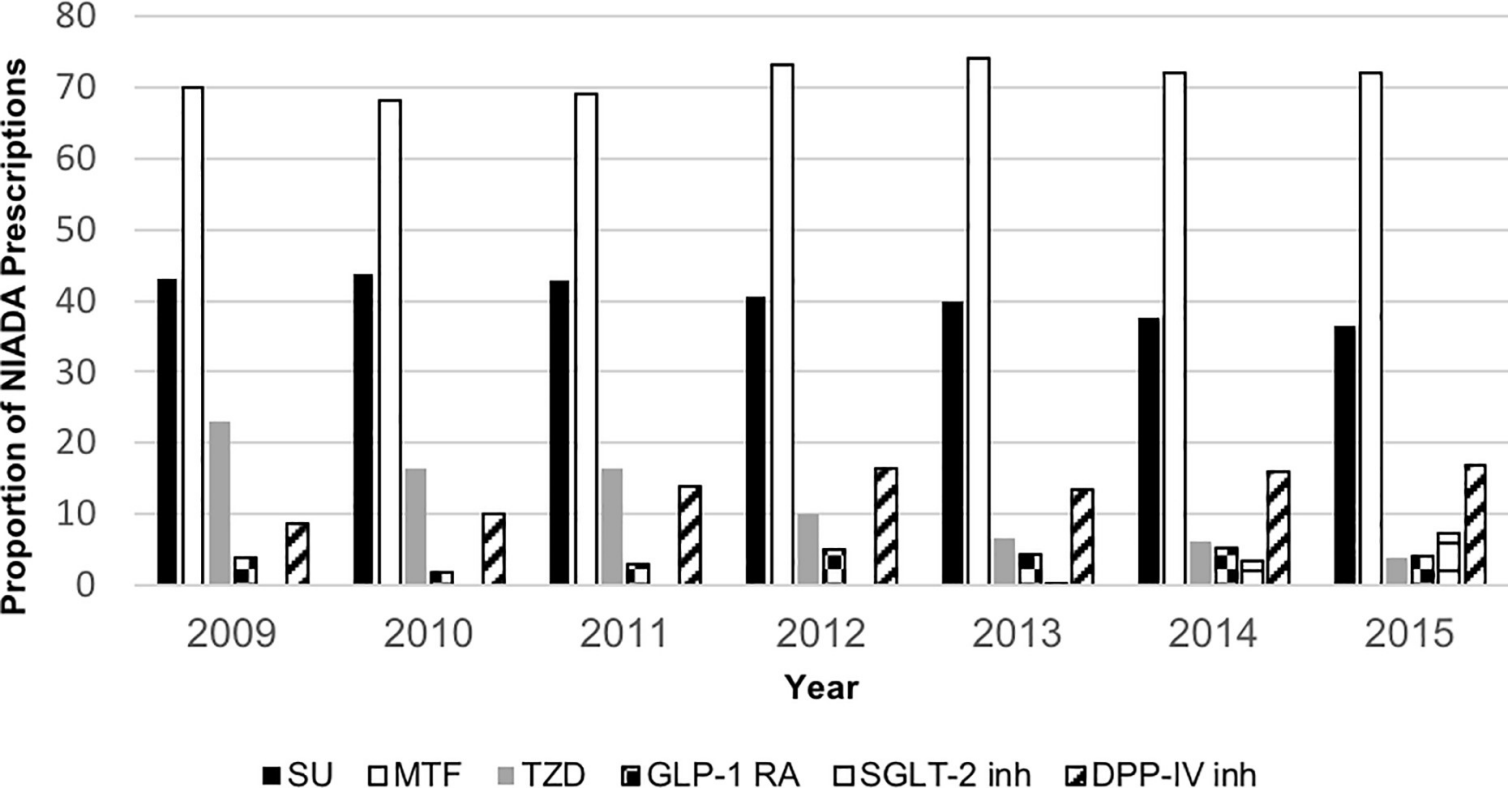
Non-insulin antidiabetic agent prescriptions 2009-2015. NIADA: Non-insulin antidiabetic agents.

**Fig 2:**
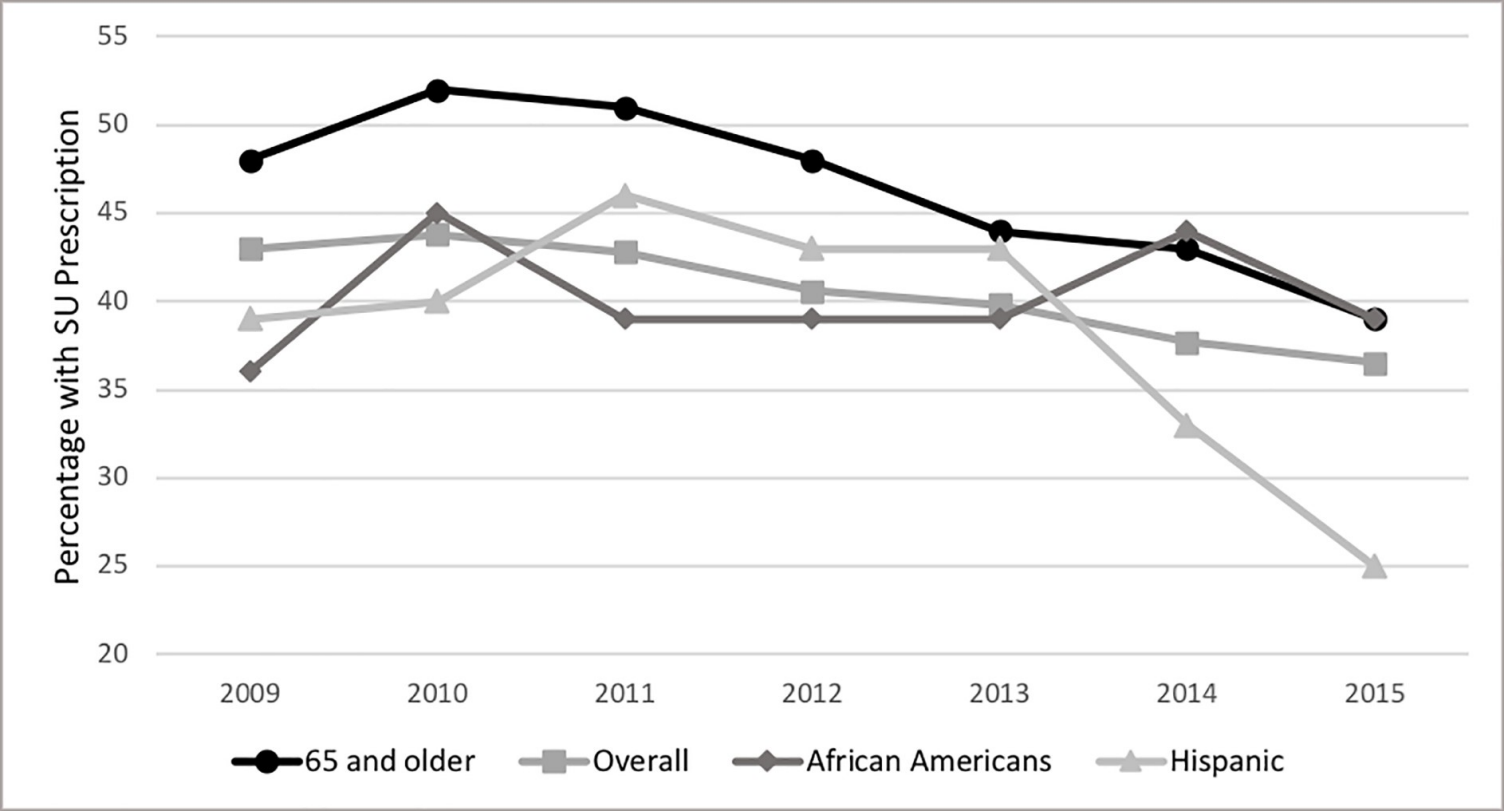
Sulfonylurea prescriptions overall and in subjects 65 or older. SU: sulfonylurea.

Sulfonylurea prescribing rates from 2009 to 2015 decreased by 6.5% (43% to 36.5%; p<0.0001) (Fig 1). In visits for patients 65 and older, sulfonylurea prescribing decreased by 9% (48% to 39%; p<0.0001) (Fig 2). Sulfonylurea prescribing in the Hispanic or Latino population decreased significantly by 13% from 2009 to 2015 (38% to 25%; p<0.0001) (Fig 2). In African Americans, sulfonylurea prescribing fluctuated from 2009 to 2015, with the lowest rate in 2009 (36%) and the highest rate in 2014 (44%) (Fig 2).

Changes in prescribing occurred across all non-insulin antidiabetic agents except metformin. Prescribing of GLP-1 RAs increased from 2009 to 2014 (4% to 5%, p<0.0001), but then decreased to 4% in 2015 (p<0.0001). SGLT-2 inhibitor prescribing began in 2013, and increased to 7% by 2015 (p<0.0001). DPP-IV inhibitor prescribing increased from 2009 to 2015 (9% to 17%; p<0.0001). TZD prescribing decreased from 2009 to 2015 (23% to 4%; p<0.0001).

Novel non-insulin antidiabetic agents were prescribed most frequently for patients with private insurance (GLP-1 RAs 57%, SGL-2 inhibitors 76%, DPP-IV inhibitors 42%), as shown in Table 2, compared to other insurance types. Metformin prescriptions were relatively stable over the seven year period (70% to 72%) (Fig 1).

**Table 2:** Insurance coverage type (in millions) for novel non-insulin antidiabetic agent prescriptions.

Insurance type	GLP-1 RA	No GLP-1 RA	SGLT-2 inhibitor	No SGLT-2 inhibitor	DPP-IV inhibitor	No DPP-IV inhibitor
Medicaid	0.4 (3%)	9.5 (3%)	0 (0%)	9.9 (3%)	0.9 (2%)	9 (3%)
Medicare	3.6 (31%)	134 (46%)	0.7 (17%)	137 (46%)	192 (47%)	118 (45%)
Private	6.7 (57%)	113 (39%)	3.3 (76%)	116 (39%)	172 (42%)	102 (39%)
Self-pay	6.9 (2%)	0.03 (0.3%)	0.04 (1%)	6.9 (2%)	0.8 (2%)	8 (3%)

## Discussion

Overall sulfonylurea prescribing decreased from 2009 to 2015, and coincided with an increase in prescriptions for GLP-1 RAs, SGLT-2 inhibitors, and DPP-IV inhibitors. The decrease in sulfonylurea prescribing is reflected across all populations; however, physicians prescribed sulfonylureas in the elderly at a higher rate than the overall population for all years, and age 65 years and older was a positive predictor of having a sulfonylurea prescription.

The decrease in sulfonylurea prescribing likely reflects providers’ attempts to minimize the occurrence of adverse events associated with sulfonylureas. In addition to causing hypoglycemia, several studies have associated sulfonylureas with increased cardiovascular risk, which stems in part from their propensity to cause weight gain and hypoglycemia [5, 15, 16]. Although sulfonylureas are one of the more effective oral anti-diabetic agents, reducing HbA1c by 1.5–2%, the HbA1c lowering effect of sulfonylureas is short-lived [17, 18]. The higher rate of sulfonylurea prescribing in the elderly is particularly concerning as sulfonylureas are one of the most common medications associated with emergency department visits for adverse drug events in individuals 65 years and older, comprising 10.7% of hospitalizations in the elderly [19]. Hospitalizations for hypoglycemia are most often associated with loss of consciousness or seizure [20]. In the elderly, loss of consciousness is more likely to lead to falls and fractures than in the general population; however, the association between sulfonylureas and falls or fractures is not well defined [21].

Frequency of TZD prescriptions also decreased from 2009 to 2015. The decline in their prescribing began in the early to mid-2000s coinciding with the identification of rosiglitazone’s association with increased cardiovascular risk [22, 23]. Although pioglitazone is not associated with increased risk of myocardial infarction (MI), and in fact demonstrates a protective effect against MI, caution is still advised since it is in the same medication class as rosiglitazone and because of its propensity to cause fluid retention and heart failure exacerbations [24]. Pioglitazone also acquired a label warning regarding thyroid c-cell tumor risk in 2011, which could have affected prescribing over the next four years [25]. Given these facts and the precipitous decrease in TZD prescribing from 23% to 3.9%, it is unlikely that TZDs played a major role in the decline in sulfonylurea use.

The decrease in sulfonylurea prescriptions corresponded with an increase in use of novel non-insulin antidiabetic agents: SGLT-2 inhibitors, GLP1-RAs, and DPP-IV inhibitors. The increase in prescriptions for these novel agents may be due to the fact that none of them independently causes hypoglycemia. Additionally, they are associated with several advantageous side effects [4]. In particular, GLP-1 RAs and SGLT-2 inhibitors can cause up to 3 kg of weight loss [26, 27]. Evidence of weight loss induced by these agents has been available since the first agents were approval by the Food and Drug Administration (FDA) in 2005 and 2013, respectively, so their relatively low prescribing prevalence is somewhat surprising. SGLT-2 inhibitors can also aid in moderately lowering blood pressure by 2 to 6 mmHg, depending on the agent [26]. Publications since 2015 have demonstrated cardiovascular benefits of SGLT-2 inhibitors and GLP-1 agonists [9–11, 28]. Unfortunately, NAMCS data post-2015 is not yet available, thus it is not possible to gauge the effect of this new information in clinical practice.

Despite the advantageous side effects and safety profiles of these novel non-insulin antidiabetic agents, their prescribing rates were still fairly low by 2015: 7.3% for SGLT-2 inhibitors, 4.2% for GLP1-RAs, and 17% for DPP-IV inhibitors. There are a number of factors that might contribute to this distribution. For example, GLP-1 RAs may represent a relatively small number of prescriptions due to patient preference for oral medications as opposed to subcutaneously administered agents; this aversion might even counteract the appeal of potential weight loss. Additionally, in 2011 GLP1-RAs received a U.S. Boxed Warning regarding increased risk of thyroid C-cell tumors [8]. Although this did not lead to a decrease in the rate of prescriptions, it probably contributed to its relative stagnancy. SGLT-2 inhibitors, on the other hand, lacked U.S. Boxed Warnings from 2009 to 2015, and prescribing rates steadily increased from 2013 to 2015. In 2015, however, all SGLT-2 inhibitors acquired warnings for increased risk of ketoacidosis, and in 2016 canagliflozin received a US Boxed Warning for increased risk of lower limb amputations [29]. Data analyzed for this manuscript do not represent the effects of these warnings on SGLT-2 inhibitor prescription rates, but future NAMCS prescription data will conceivably reflect providers’ response to these warnings. With respect to both agents, use necessitates vigilant screening for these adverse drug events prior to initiation. Additionally, their relative high cost affects who can reasonably be prescribed those drugs, evidenced by the fact that they are most often prescribed in patients with private insurance and least often prescribed in those who self-pay.

In spite of the overall decrease in sulfonylurea prescription rates, sulfonylurea prescribing remained unduly high by 2015, at 36.5%. Perhaps the most compelling explanation for the more frequent use of sulfonylureas over novel non-insulin agents is efficacy. On average, GLP1-RAs, SGLT-2 inhibitors, and DPP-IV inhibitors lower HbA1c by 0.5% to 1%, 0.5% to 1.2%, and approximately 0.8%, respectively, as opposed to the 1.5% to 2% lowering of sulfonylureas [3, 17]. Sulfonylureas are an inexpensive, oral, effective option for lowering HbA1c in the short term. Additionally, from 2009 to 2015, the ADA Standards of Care guidelines offered little direction regarding selection of an add-on antidiabetic agent [30–36]. While they clearly designated metformin as first-line therapy, they did not specify a systematic process for choosing between other available antidiabetic agents for add-on therapy. The 2015 guidelines recommend choosing an add-on medication based on patient preference, drug characteristics, and avoidance of adverse effects, especially hypoglycemia [36]. This differs significantly from the 2019 ADA Standards of Care guidelines, which provide an algorithm for choosing add-on medications based on patient-specific comorbidities and specifies that hypoglycemia should be avoided in older adults [37].

The more thorough guidelines available in 2019 will aid clinicians in considering the risk to benefit ratio for an individual patient when deciding between non-insulin antidiabetic agents. Namely, all three novel antidiabetic medication classes allow for blood glucose lowering without hypoglycemia, and SGLT-2 inhibitors and GLP1-RAs are associated with weight loss. Additionally, the novel agents alter the underlying pathophysiology of DM while sulfonylureas do not [7]. Considering the benefits of the newer non-insulin antidiabetic agents, sulfonylureas will likely have a more limited role in the treatment of type 2 diabetes. They may be a useful option for patients who struggle with the cost of medications. In patients with private insurance, however, the cost of at least one medication per class is usually covered, deeming the prescribing of certain agents for the sake of cost unnecessary.

This study is limited by its retrospective study design. Only the first eight medications listed per outpatient visit were included in this study, potentially resulting in falsely low prescribing rates. However, unaccounted medications would be similar across study years and not affect national trends derived from NAMCS. In addition, at the time of this analysis 2015 survey results were the most recently available, whereas studies published in 2016 revealed the cardiovascular benefit of SGLT-2 inhibitors and GLP-1 agonists. Thus, this study was unable to analyze the impact of these studies on non-insulin antidiabetic medication prescribing trends.

## Conclusions

In conclusion, sulfonylurea prescribing continually decreased from 2009 to 2015, likely due to increasing recognition of their adverse effects and availability of novel non-insulin antidiabetic agents. However, prescribing of sulfonylureas remains highest in elderly patients, who are most at risk for experiencing adverse effects. Novel non-insulin antidiabetic agents, especially SGLT-2 inhibitors and GLP1-RAs, offer substantial benefits, including weight loss and alteration of the underlying pathophysiology of type 2 diabetes, but also require screening and monitoring for adverse drug events. Decreased prescribing of sulfonylureas and increased prescribing of novel non-insulin antidiabetic agents will like likely continue in the coming years as generic forms of novel medications become available.

## Supporting information

S1 File NAMCS NIADA prescribing data 2009–2015. NAMCS: National Ambulatory Medical Care Survey. NIADA: Non-insulin antidiabetic agents.
(CSV)
